# Identifying Alzheimer’s genes via brain transcriptome mapping

**DOI:** 10.1186/s12920-022-01260-6

**Published:** 2022-05-19

**Authors:** Jae Young Baik, Mansu Kim, Jingxuan Bao, Qi Long, Li Shen, Alzheimer’s Disease Neuroimaging Initiative

**Affiliations:** 1 School of Arts and Sciences, University of Pennsylvania, Philadelphia, USA; 2 Department of Artificial intelligence, Catholic University of Korea, Bucheon, Republic of Korea; 3 Department of Biostatistics, Epidemiology and Informatics, Perelman School of Medicine at the University of Pennsylvania, Philadelphia, USA

**Keywords:** Brain imaging transcriptomics, Imaging-diagnosis map, Gene expression map

## Abstract

**Background:**

Alzheimer’s disease (AD) is one of the most common neurodegenerative disorders characterized by progressive decline in cognitive function. Targeted genetic analyses, genome-wide association studies, and imaging genetic analyses have been performed to detect AD risk and protective genes and have successfully identified dozens of AD susceptibility loci. Recently, brain imaging transcriptomics analyses have also been conducted to investigate the relationship between neuroimaging traits and gene expression measures to identify interesting gene-traits associations. These imaging transcriptomic studies typically do not involve the disease outcome in the analysis, and thus the identified brain or transcriptomic markers may not be related or specific to the disease outcome.

**Results:**

We propose an innovative two-stage approach to identify genes whose expression profiles are related to diagnosis phenotype via brain transcriptome mapping. Specifically, we first map the effects of a diagnosis phenotype onto imaging traits across the brain using a linear regression model. Then, the gene-diagnosis association is assessed by spatially correlating the brain transcriptome map with the diagnostic effect map on the brain-wide imaging traits. To demonstrate the promise of our approach, we apply it to the integrative analysis of the brain transcriptome data from the Allen Human Brain Atlas (AHBA) and the amyloid imaging data from the Alzheimer’s Disease Neuroimaging Initiative (ADNI) cohort. Our method identifies 12 genes whose brain-wide transcriptome patterns are highly correlated with six different diagnostic effect maps on the amyloid imaging traits. These 12 genes include four confirmatory findings (i.e., AD genes reported in DisGeNET) and eight novel genes that have not be associated with AD in DisGeNET.

**Conclusion:**

We have proposed a novel disease-related brain transcriptomic mapping method to identify genes whose expression profiles spatially correlated with regional diagnostic effects on a studied brain trait. Our empirical study on the AHBA and ADNI data shows the promise of the approach, and the resulting AD gene discoveries provide valuable information for better understanding biological pathways from transcriptomic signatures to intermediate brain traits and to phenotypic disease outcomes.

**Supplementary Information:**

The online version contains supplementary material available at 10.1186/s12920-022-01260-6.

## Background

Alzheimer’s disease (AD) is one of the most common neurodegenerative disorders characterized by a decline in cognitive function. Given that many studies demonstrated the high heritability of AD, ranging from 60 to 80%, researchers have performed targeted genetic studies or genome-wide association studies (GWAS) to link genetic variants such as single nucleotide polymorphisms (SNPs) to clinical outcomes [1].

These conventional genetic association studies discovered many AD-related genetic variants, including those from genes such as apolipoprotein E (APOE), clusterin (CLU), ATP-binding cassette, sub-family A (ABC1), member7 (ABCA7), complement component(3b/4b) receptor1 (CR1), and others [2–4]. However, it remains a challenge to interpret and validate these SNP-diagnosis associations directly since there is a lack of intermediate molecular signatures (e.g., epigenomic, transcriptomic, proteomic, and metabolomic measures) and system-level biomarkers (fluid biomarkers, brain structural and functional measures, and cognitive and behavioral measures) to link genetics to disease status [5–7].

Recent studies have investigated the associations between molecular features (e.g., gene expression) with disease-related imaging traits [8–11]. The assays of gene expression, in particular, offer a unique opportunity to measure gene functions. The Allen Human Brain Atlas (AHBA) provides us comprehensive whole-genome whole-brain microarray transcriptomics data. Many researchers have documented molecular and cellular processes as well as the brain structural and functional changes involved in neurodegeneration by measuring spatial correlations between gene expression patterns and brain imaging phenotypes. For example, prior studies employed a correlation or regression model to link neuroimaging features (e.g., transverse relaxation time and regional atrophy) and transcriptomics data [8, 12–14]. These imaging transcriptomic studies often did not directly involve the diagnostic outcome in the analyses. Thus, the identified imaging or transcriptomic markers might not be related or specific to disease outcomes such as AD.

**Fig. 1:**
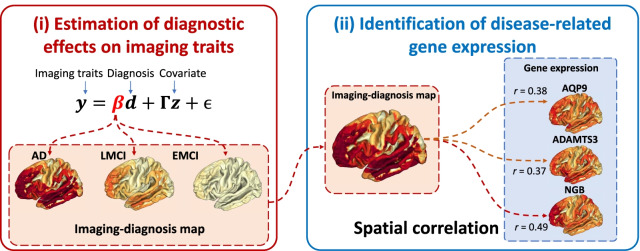
The overall workflow for identifying novel genes related to Alzheimer’s Disease (AD). The workflow involves: (i) estimating the diagnostic effects on the image traits across the brain using a linear regression model; and (ii) identifying AD-specific genes via spatially correlating imaging-diagnosis map with gene expression map in the brain

In this study, we hypothesize that the genes whose expression patterns in the brain spatially correlated with the diagnostic pattern of AD-related neuroimaging traits, like beta-amyloid deposition, will also be associated with AD. Thus, we propose an innovative two-stage approach to identify the disease-related genes by correlating spatial gene expression map with regional diagnostic effects on imaging traits in the brain (Fig. 1). We demonstrate the promise of our approach to identifying disease-related gene expression signatures using the brain transcriptome data from the AHBA [15] and the amyloid imaging data from the Alzheimer’s Disease Neuroimaging Initiative (ADNI) cohort [16]. Our approach identifies not only multiple confirmatory findings (i.e., AD genes from the DisGeNET database [17]) but also numerous novel genes associated with various disease stages (i.e., early mild cognitive impairment [EMCI], late mild cognitive impairment [LMCI], and AD); and these novel genes have not been previously linked to AD in DisGeNET.

## Results

In this section, we present the results of our empirical study with the ADNI and AHBA data to demonstrate the promise of our approach. Briefly, in our stage 1 regression analysis, we noted that cognitive normal (CN) vs AD participants yielded the most significant diagnostic effects on the AV45 imaging traits, while CN vs EMCI yielded the least significant ones. In our stage 2 correlation analysis, we identified 12 genes, including 4 AD-related and 8 non-AD-related genes based on the DisGeNET database, whose gene expression maps in the brain were spatially correlated with all the six diagnostic effect maps on AV45 imaging traits. See below for more detailed description of these results.

### Mapping diagnostic effects on the AV45 imaging traits across the brain via linear regression

**Fig. 2:**
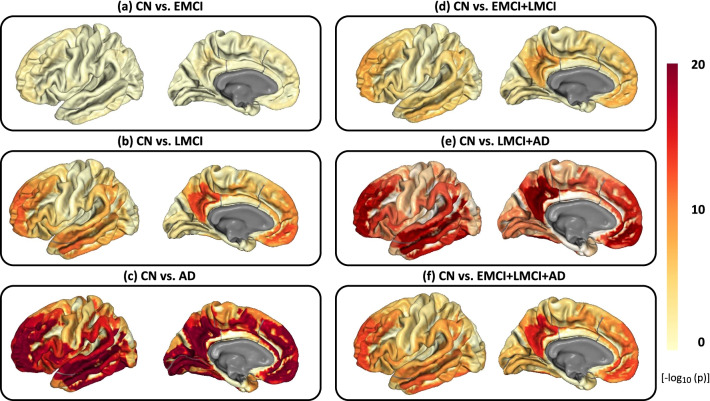
Brain maps of imaging-diagnosis associations. **a**–**f** The brain maps for six different case-control comparisons respectively. The $-log_{10}(p)$ values for each analysis are color-coded and mapped onto the brain

We created an imaging-diagnosis map for each of six diagnostic comparisons through applying a linear regression model to capture the diagnostic effects on AV45 imaging traits across the brain. Figure 2 shows all six brain maps of imaging-diagnosis associations for six different case-control comparisons respectively. The $-log_{10}(p)$ values for each analysis are color-coded and mapped onto the brain. We observed that CN vs AD yielded the most significant diagnostic effects on the AV45 imaging traits, while CN vs EMCI yielded the least significant ones (Table 1).

**Fig. 3:**
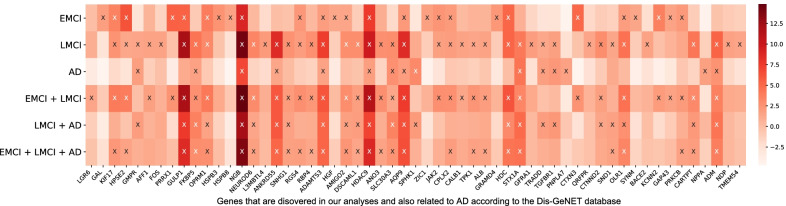
The heat map of significant gene findings that are related to AD according to Dis-GeNET. The heat map includes all the genes that are discovered in our analyses and also related to AD according to the Dis-GeNET database. The six diagnostic comparisons on the y axis are plotted against all the genes on the x-axis. The $-log_{10}(p)$ values are color-coded and shown in the heat map. The symbol “X” denotes significant correlataion between the imaging-diagnosis map and the gene expression map (corrected p < 0.05)

**Fig. 4:**
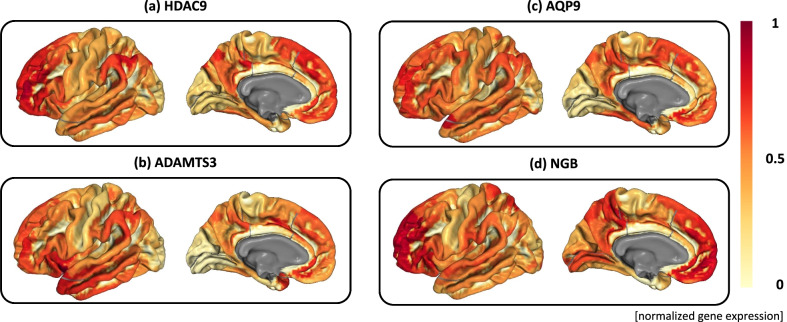
Gene expression level on the brain for the identified AD-related genes. **a**–**d** show the brain transcriptome maps for four AD-related genes (i.e., AQP9, ADAMTS3, HDAC9, and NGB) respectively. The normalized gene expression level is color-coded and mapped onto the brain. These maps are significantly correlated with imaging-diagnosis maps for all six case-control comparisons shown in Fig. 2

**Fig. 5:**
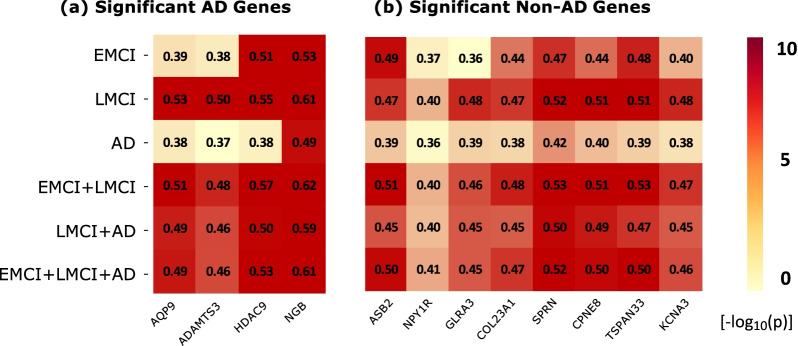
The heat maps of 12 significant gene findings that are correlated to all six diagnostic comparisons. **a** 4 genes that are related to AD according to DisGeNET, and **b** 8 genes that are not related to AD according to DisGeNET. The $-log_{10}(p)$ values are color-coded and shown in the heat maps. The correlation coefficients are shown in each cell

**Table 1:** Demographic information

	CN	EMCI	LMCI	AD	Total
Number of subject	255	296	218	202	971
Age	76.35 ± 6.54	71.78 ± 7.28	74.71 ± 8.39	75.85 ± 7.67	74.48 ± 7.67
Sex (male/female)	132/123	167/129	129/89	123/79	551/420
Education (year)	16.37 ± 2.64	12.12 ± 2.64	16.12 ± 2.94	15.83 ± 2.81	16.13 ± 2.75

**Table 2:** Top 10 significant regions associated with diagnostic outcome

ROI	Network	CN versus EMCI	CN versus LMCI	CN versus AD	CN versus EMCI + LMCI	CN versus LMCI + AD	CN versus EMCI + LMCI + AD
L_10pp_ROI	Posterior-multimodal	3.21			8.08		
L_10r_ROI	Posterior-multimodal		12.86	28.06		20.61	
L_10v_ROI	Posterior-multimodal	2.15					
L_31a_ROI	Posterior-multimodal	2.05	12.63		8.48	20.60	14.36
L_31pd_ROI	Posterior-multimodal		11.98	29.56		22.35	13.89
L_31pv_ROI	Posterior-multimodal		12.31	30.03		22.48	13.48
L_6ma_ROI	Cingulo-opercular	2.03					
L_7m_ROI	Posterior-multimodal		12.76	30.00	7.96	22.92	14.51
L_9-46d_ROI	Cingulo-opercular				7.79		13.53
L_9a_ROI	Posterior-multimodal	2.54			8.48		
L_9p_ROI	Posterior-multimodal	2.42			7.81		
L_a10p_ROI	Frontoparietal	2.03					
L_a47r_ROI	Frontoparietal	3.00					
L_d23ab_ROI	Posterior-multimodal		13.96	32.99	8.76	24.26	15.72
L_FFC_ROI	Visual2			30.42		21.20	
L_p32_ROI	Posterior-multimodal		12.64				
L_p47r_ROI	Frontoparietal	2.48			7.99		13.22
L_PCV_ROI	Dorsal-attention	3.15	12.91		9.55	21.68	15.79
L_POS1_ROI	Posterior-multimodal			37.73		22.03	
L_s32_ROI	Posterior-multimodal		12.53		7.89		13.33
L_TE2p_ROI	Language			29.02			
L_v23ab_ROI	Posterior-multimodal		12.75	37.54		24.64	14.89
L_VVC_ROI	Visual2			33.08			

The significance level is reported in the format of $-log_{10}(p)$-value. The network is annotated to the each ROI according to the Cole-Anticevic Brain-side Network [32]

Shown in Additional File 1: Table S1 is all the significant ROI-diagnosis associations across the six experiments. In total, 171 out of 180 ROIs were identified to be associated with at least one diagnostic outcome. In addition, 39 ROIs were significantly associated with all six diagnostic outcomes. In detail, 40 ROIs are significant for CN vs EMCI, 145 ROIs are significant for CN vs LMCI, 169 ROIs are significant for CN vs AD, 134 ROIs are significant for CN vs EMCI+LMCI, 167 ROIs are significant for CN vs LMCI+AD, and 162 ROIs are significant for CN vs EMCI+LMCI+AD. These findings are in accordance with our knowledge, where the abnormality of AV45 imaging traits gradually increases over the disease progression from EMCI to LMCI and then to AD.

Table 2 shows the top ten most significant ROIs across the six different diagnostic comparisons. Among these top findings, there are in total 23 ROIs with significant diagnostic effects on the AV45 imaging traits in one or more diagnosttic comparisons; and 14 out of 23 ROIs (i.e., L_10pp_ROI, L_10r_ROI, L_10v_ROI, L_31a_ROI, L_31pd_ROI, L_31pv_ROI, L_7m_ROI, L_9a_ROI, L_9p_ROI, L_d23ab_ROI, L_p32_ROI, L_PO S1_ROI, L_s32_ROI, and L_v23ab_ROI) are subregions of the bilateral dorsomedial parietal lobe, temporal-parietal-occipital junction, and dorsocaudal temporal lobe. Three ROIs (i.e., L_a10p_ROI, L_a47r_ROI, and L_p47r_ROI) are subregions of the frontoparietal cognitive control network.

### Identifying disease-related genes through brain transcriptome mapping using correlation analysis

We applied Pearson correlation analysis to identify disease-related genes whose expression maps were spatially correlated with the imaging-diagnosis maps. In total, 295 unique genes were identified across the six experiments, and we summarized all these results in Additional File 1: Table S2(a). Specifically, CN vs AD yielded the smallest number of significant correlations with only 40 significant genes, and CN vs LMCI+AD yielded the largest number of significant genes with 214 significant genes.

Out of 295 significant genes, 57 genes have already been linked to AD according to the DisGeNET database [17], and we summarized these 57 gene findings in Additional File 1: Table S2(b). Figure 3 shows the heat map of these 57 gene findings, where the $-log_{10}(p)$ values of diagnosis-by-gene correlations are shown between the six diagnostic comparisons on the y axis and all 57 genes on the x-axis. There are four genes (i.e., AQP9, ADAMTS3, HDAC9, and NGB) whose expression maps are significantly correlated with imaging-diagnosis maps for all six diagnostic comparisons. Figure 4 shows the gene expression profiles of the above four genes mapped onto the brain. A visual comparison between six diagnostic effect maps in Fig. 2 and four gene expression maps in Fig. 4 indicates that the spatial patterns of these maps are strongly correlated.

Figure 5a shows the heat map of four significant gene findings that are not only correlated to all six diagnostic comparisons but also are related to AD according to the DisGeNET database. It is noted that the NGB gene expression pattern has the highest correlations with the imaging-diagnosis maps across the six comparisons (i.e., 0.53 for CN vs EMCI, 0.61 for CN vs LMCI, 0.49 for CN vs AD, 0.62 for CN vs LMCI+AD, 0.59 for CN vs LMCI+AD, and 0.61 for CN vs EMCI+LMCI+AD), and the ADAMTS3 gene expression pattern exhibits the lowest correlations (i.e., 0.38 for CN vs EMCI, 0.50 for CN vs LMCI, 0.37 for CN vs AD, 0.48 for CN vs LMCI+AD, 0.46 for CN vs LMCI+AD, and 0.46 for CN vs EMCI+LMCI+AD).

Out of the 295 genes, there are 238 genes that have not been linked to AD in DisGeNET, denoted as “non-AD-genes”. These 238 gene findings are shown in Additional File 1: Table S2(c). Among these 238 genes, there are 8 genes (i.e., ASB2, NPY1R, GLRA3, COL23A1, SPRN, CPNE8, TSPAN33, and KCNA3) whose expression maps are significantly correlated with imaging-diagnosis maps for all six diagnostic comparisons. These findings suggest potential connections between these genes and AD through underlying transcriptomic and/or amyloid mechanisms in the brain, which is an interesting research topic warranting further investigation. Figure 5b shows the heat map of these 8 significant gene findings. The SPRN gene has the highest correlation coefficients (i.e., 0.47 for CN vs EMCI, 0.52 for CN vs LMCI, 0.42 for CN vs AD, 0.53 for CN vs LMCI+AD, 0.50 for CN vs LMCI+AD, and 0.52 for CN vs EMCI+LMCI+AD), and the NPY1R gene has the lowest correlation coefficients (i.e., 0.37 for CN vs EMCI, 0.40 for CN vs LMCI, 0.36 for CN vs AD, 0.40 for CN vs LMCI+AD, 0.40 for CN vs LMCI+AD, and 0.41 for CN vs EMCI+LMCI+AD).

Among 12 gene findings shown in Fig. 5a, b, we observed that CN vs AD yielded the lowest correlation coefficients for all 12 genes, in comparison with all the other five diagnostic paradigms. This pattern appears intriguing, and warrants replication in independent cohorts as well as further investigation for possible mechanistic understanding.

## Discussion

In this work, we have proposed a two-stage approach to link transcriptome data to diagnosis outcome via brain mapping and have applied it to the AHBA and ADNI data to demonstrate its promise. Many studies have performed correlation or regression analysis to investigate the relationship among genetics, transcriptomics and neurodegenerative disorders [7]. To the best of our knowledge, this work is the first study to link diagnostic outcomes and transcriptomic data via human brain mapping. Across the six experiments, we confirmed the following four genes that are linked to AD in the DisGeNET database: AQP9, ADAMTS3, HDAC9, and NGB, as shown in Fig. 5a. In addition, we identified the following eight novel genes not yet linked to AD according to DisGeNET: ASB2, NPY1R, GLRA3, COL23A1, SPRN, CPNE8, TSPAN33, and KCNA3, as shown in Fig. 5b.

We observed that four significant AD genes were correlated with all six case-control comparisons—AQP9, ADAMTS3, HDAC9, and NGB. AQP9 was reportedly expressed in the neurons of the substantia nigra, tanycytes, and some astrocytes [18]. One mouse study demonstrated that the reduction of ADAMTS3 contributed to the inhibition of amyloid $\beta$ decomposition [19]. One study demonstrated that HDAC9 was reported to be part of the pathway that mediates synaptic function and amyloid precursor protein processing in AD [20]. The expression of NGB was also reported to be down-regulated with increasing age, down-regulated in women (consistent with their increased risk), and up-regulated in the temporal lobe of AD patients (consistent with a response to the disease process) [21]. All these existing findings collectively support our discoveries and demonstrate the effectiveness of our method in identifying AD-related genes supported by the transcriptomic evidence in the brain.

Additionally, we observed that eight non-AD genes significantly correlated with the image-diagnosis map for all six case-control comparisons—ASB2, NPY1R, GLRA3, COL23A1, SPRN, CPNE8, TSPAN33, and KCNA3. We used the statistical over-representation test of PANTHER [22] to detect statistical over-representation of the eight significant non-AD genes compared to the human genome reference gene list. In this process, the enriched biological process of Homo sapiens species via gene ontology (GO) [23, 24] was identified. The results showed that 28 GO biological processes passed the nominal threshold of $p < 0.05$. Among all these significant GO biological processes, “neuropeptide signaling pathway” was enriched with the smallest raw p-value $1.01\times 10^{-3}$ and with fold enrichment 41.99. Existing findings, including apeline, Neuropeptide Y, and dynorphin A, which were important neuropeptides, were closely related to AD [25–27]. Moreover, evidence showed that in AD patients and AD animal models, numerous neuropeptide-containing neurons were pathologically altered in their brain areas [28]. The levels of various neuropeptides had also been found altered in both Cerebrospinal Fluid, and blood of AD patients [28]. All these existing findings demonstrate an important role of “neuropeptide signaling pathway” played in AD, indicating that the eight significant DisGeNET non-AD genes might have a connection to AD. It warrants further investigation to evaluate the potential of these genes as AD related genes.

Many AD studies demonstrated that the accumulation of misfolded amyloid $\beta$ and tau protein causes degeneration and loss of neuronal function in the brain [29, 30]. Our analyses yielded promising gene findings through mapping gene expression pattern and AD-related amyloidosis pattern in the brain. Our study not only confirmed known AD genes but also suggested novel gene targets with potential to be linked to AD through underlying transcriptomic and amyloid mechanisms in the brain. These discoveries provide valuable information to guide further neurobiological and molecular studies to reveal the biological pathway from gene expression to amyloid accumulation and to the degeneration and loss of neuronal function in the brain.

## Conclusion

We have proposed a two-stage approach to identify genes whose expression levels are related to diagnosis phenotype via brain transcriptome mapping. Specifically, we first mapped the effects of a diagnosis phenotype onto imaging traits across the brain using a linear regression model. Then, the gene-diagnosis association was assessed by spatially correlating the brain transcriptome map with the diagnostic effect map on the brain-wide imaging traits. We applied our approach to analyze the brain transcriptome data from the AHBA and the amyloid imaging data from the ADNI cohort to demonstrate the promise of our approach. Our approach detected four genes with AD link shown in DisGeNET and eight genes that have not yet been linked to AD in DisGeNET. Our proposed novel disease-related brain transcriptomic mapping method was designed to spatially associate gene expression profiles with regional diagnostic effects on a brain trait to reveal disease-related genes. Our empirical study on the AHBA and ADNI data shows the promise of the approach. The resulting AD gene discoveries provide valuable information to better understand biological pathways from transcriptomic signatures to intermediate brain traits and phenotypic disease outcomes.

## Methods

### Data description

Data used in the preparation of this article were obtained from the ADNI database (adni.loni.usc.edu) [16]. The ADNI was launched in 2003 as a public-private partnership, led by Principal Investigator Michael W. Weiner, MD. The primary goal of ADNI has been to test whether serial magnetic resonance imaging (MRI), positron emission tomography (PET), other biological markers, and clinical and neuropsychological assessment can be combined to measure the progression of mild cognitive impairment (MCI) and early Alzheimer’s disease (AD). For up-to-date information, see www.adni-info.org.

In this study, we included a total of 971 participants (i.e., 255 Cognitive Normal [CN], 296 EMCI, 218 LMCI, and 202 AD subjects) who had complete [$^{18}$F]florbetapir (AV45) PET data (measuring amyloid burden), and diagnostic and clinical assessments. The detailed demographic information is presented in Table 1. For AV45 PET scans, the data was registered to the Montreal Neurological Institute space, and the standard uptake value ratio was computed by intensity normalization using the cerebellar curs reference region. Region-level AV45 measures were extracted based on the human connectome project [31] multi-modal parcellation (HCPMMP) atlas, consisting of 360 regions of interests (ROIs).

The brain-wide transcriptome (gene expression) data was downloaded from the Allen Human Brain Institute [15]. The AHBA offered the gene expression levels of more than 20,000 genes from 3,702 distinct tissues, which were sampled across six different donors to cover the entire brain. Of these six honors, only two donors had tissue samples from both hemispheres, and the remaining four donors had the samples only from the left hemisphere. In this work, we preprocessed the AHBA data using the pipeline proposed by Aurina *et al.* and mapped those onto the HCPMMP atlas [8]. After that, we focused our analyses on the data from the left hemisphere (including 180 ROIs), since it is more densely sampled than the right one.

### Two-stage method for identifying novel genes related to Alzheimer’s disease

Here we propose a two-stage method for identifying novel genes related to Alzheimer’s disease, as summarized in Fig. 1. In the first stage, we perform linear regression to estimate the diagnostic effects on the image traits across the brain. In the second stage, we identify novel genes whose gene expression levels in the brain are spatially correlated with the diagnostic effects on the imaging traits across the brain.

#### Stage 1. Regression analysis to estimate diagnostic effect on the AV45 imaging traits in the brain: imaging-diagnosis map

Utilizing the AV45 imaging traits and diagnostic phenotype, we performed linear regression analysis to estimate the diagnostic effect on imaging traits. In this analysis, we used the ROI-based imaging trait as the response, the diagnostic phenotype (i.e., control vs case) as the predictor, and age, sex, and education level as covariates to remove confounding effects. Since AD is a progressive neurodegenerative disorder, it is important to perform the analysis for different disease stages, where the disease progresses from EMCI to LMCI and then to AD (i.e., EMCI < LMCI < AD). With this observation, we examined the following six diagnostic comparisons: 1) CN vs EMCI, 2) CN vs LMCI, 3) CN vs AD, 4) CN vs EMCI+LMCI, 5) CN vs LMCI+AD, and 6) CN vs EMCI+LMCI+AD. Of note, CN vs EMCI+AD comparison was excluded due to the stage discontinuity between EMCI and AD (i.e., LMCI is in-between). We applied our approach to each of the above six cases to estimate the corresponding diagnostic effect on the brain traits. As a result, we generated the significance map (imaging-diagnosis map) to assess the diagnostic effects in the brain. The significance was reported in format of $-\hbox {log}_{10}(p\text{-value })$. We then excluded all the ROIs in the right hemisphere, to solely focus on the left hemisphere for the reason we stated earlier.

#### Stage 2. Correlation analysis between gene expression map and imaging-diagnosis map

In this section, we identify disease-related genes whose expression levels are spatially correlated to imaging-diagnosis map through computing their Pearson’s correlation coefficients. We performed this analysis 60,162 times (i.e., 6 imaging-diagnosis maps $\times$ 10,027 genes expression maps from the AHBA database) and the Bonferroni method was applied for multiple comparison correction. Due to the preprocessing step for the AHBA data, we only utilized the left hemisphere (i.e., 180 ROIs out of the total 360 ROIs) for both imagining-diagnosis map and the AHBA transcriptome map. In addition, we further analyzed the identified genes to examine their associations with AD based on the DisGeNET database [17]. The DisGeNET’s comprehensive database for disease association studies was utilized to assess whether or not the significant genes that we have identified have been associated with AD in prior studies. In DisGeNET, there are 3397 genes related to “Alzheimer’s Disease” (UMLS CUI: C0002395). Out of these 3397 genes, we only looked at a subset of 1877 genes since the pre-processing step for the AHBA data removed genes with insignificant expression.

## Supplementary Information

**Additional file 1. Table S1**: Significance level of brain regions using imaging-diagnosis analysis. **Table S2(a)**: Significance level of identified genes significantly associated with diagnostic outcome using correlation analysis. **Table S2(b)**: Significance level of identified genes related with AD based on DisGeNET. **Table S2(c)**: Significance level of identified genes unrelated with AD based on DisGeNET.

## Data Availability

The datasets used and analyzed during the study are available in the ADNI LONI repository and Allen brain atlas data portal, https://adni.loni.usc.edu/ and https://portal.brain-map.org/, respectively.

## Abbreviations

AD: Alzheimer’s disease
ADNI: Alzheimer’s disease neuroimaging initiative
ROI: Region of interest
AHBA: Allen Human Brain Atlas human brain microarray data
AV45: [^18^F]florbetapir
EMCI: Early mild cognitive impairment
LMCI: Late mild cognitive impairment
CN: Cognitively normal
